# Forecasting the volatility of educational firms based on HAR model and LSTM models considering sentiment and educational policy

**DOI:** 10.1016/j.heliyon.2024.e38560

**Published:** 2024-09-26

**Authors:** Xuefan Li, Donghua Li, Yuxiang Cheng, Wen Li

**Affiliations:** a Ontario Institute for Studies in Education, University of Toronto, Toronto, Ontario, Canada; b Canada City High School, Toronto, Ontario, Canada; c School of Economics, Peking University, Beijing, China; d School of Public Administration, Southwestern University of Finance and Economics, Chengdu, China

**Keywords:** Education stock volatility prediction, Investor sentiment, Political policy, LSTM model, HAR model

## Abstract

This study aims to investigate the impact of sentiment and policy on the volatility of educational stock prices by using HAR (Heterogeneous Auto Regressive) and LSTM (Long Short-Term Memory) models. We construct a weighted educational index volatility composed of nine publicly traded educational companies from the Shenzhen Stock Exchange and Shanghai Stock Exchange, and analyze the impact of sentiment and policy variables on the volatility of educational stock prices. We use OLS regression models and LSTM prediction models to analyze the data by developing various of models to investigate the impact of sentiment, education policies and their intersection effect. The empirical results show that the sentiment index and policy index have significant impacts on different time horizons of educational stock price volatility. The LSTM model confirms the effectiveness of including sentiment and policy variables in predicting educational stock price volatility. These findings carry several practical implications, particularly for investors, education-listed companies, and policymakers. And this study contributes to the literature by providing new evidence on the impact of sentiment and policy on the volatility of educational stock prices and by demonstrating the usefulness of combining HAR and LSTM models in predicting stock price volatility.

## Highlights

• Investigate the impact of sentiment and policy on the volatility of educational stock prices.
• Construct a weighted educational index comprised of educational companies.
• Conduct a dictionary-based sentiment analysis to determine the sentiment index.
• The LSTM model confirms the effectiveness of including sentiment and policy variables in predicting educational stock price volatility.

## 1 Introduction

In recent years, the Internet has developed rapidly, and many traditional industries have continued to innovate to gain profits in the process of Internet economic development. New business models have emerged one after another, and online education companies have risen rapidly. With more and more online education companies, its economic activities and capital markets have shown explosive growth. Investment, mergers and acquisitions, restructuring and other economic activities among education companies are becoming more and more frequent.

The education industry is one of the most important components of the economic development of a country. The education industry in China has been growing rapidly, attracting the attention of investors. However, the volatility of educational stock prices poses a significant risk to investors, making volatility prediction a crucial task for effective investment decision-making. Previous studies have shown that sentiment and policy variables can impact stock price volatility. Lots of literature finds that investor sentiment leads to a dramatic fluctuation in listing firms’ stock prices [1,2].

Besides, the education industry is also very affected by political policies [3]. For example, in 2019, the Covid-19 broke out in an all-round way. In order to ensure the prevention and control of the epidemic, China announced the policy of “suspending classes without stopping learning”. The offline education that has long been a common teaching model has been completely suspended. Online education has become the mainstream teaching choice during the pandemic. This situation leads to prosperity and volatility in the share prices of online education companies. Moreover, In July 2021, the Chinese government promulgated a double reduction policy to reduce the burden on family education [4]. This policy sets clear restrictions on discipline-based training institutions, which can prevent the excessive expansion and monopoly of the capital market. It has greatly affected the future cash flow income of education companies. This also caused panic in the education capital market, and education and training stocks fell sharply. Scholar Education fell by 45 %, New Oriental-S fell by 47 %, and Excellence Education Group fell by 42 %. In the A-share market, DouShen Education fell by 20 %, and Qinshang, Xueda Education, and Only Education fell by their limit. In the U.S. stock market, Zhonggai education stocks fell across the board, among which TAL closed down 70 % and Gaotu closed down 63 % [5]. To capture the volatility information of education listing firms, it is crucial to incorporate investor sentiment and political policies in the stock market predicting.

The stock market is one of the most dynamic and complex financial markets. Understanding the factors that affect stock price volatility has been a long-standing research topic in finance for decades. Various machine learning and time series models have been applied to predict stock prices and their volatility. However, most of the work usually considers low-frequency stock price information in the linear forecasting model, such as ARIMA model, GARCH model, and VAR model. The HAR model can capture the heterogeneous influence of different types of traders in the market based on high-frequency data, which provides a more accurate prediction [6,7]. LSTM model as a nonlinear model is always utilized to predict stock price, which is proven to have a more accurate performance [8]. However, few studies have incorporated both HAR and LSTM models into the analysis to predict the volatility of educational stock prices.

Against this background, this study aims to investigate the impact of sentiment and policy on the volatility of educational stock prices. Specifically, we will construct a weighted educational index volatility comprised nine publicly traded educational companies from the Shenzhen Stock Exchange and Shanghai Stock Exchange, and analyze the impact of sentiment and policy variables by using both HAR and LSTM models.

This study collects the 5-min trading data of nine public companies listed on the Shanghai Stock Exchange and Shenzhen Stock Exchange, and constructs a weighted average educational index volatility for prediction. Sentiment and policy variables are extracted through sentiment analysis and policy announcements. The study then uses HAR models to identify the impact of sentiment and policy on the volatility of educational stock prices and LSTM models to predict volatility.

The empirical results of this study show that the sentiment index has a significant impact on the daily and weekly volatility of educational stock prices, while the policy index has a significant impact on the monthly volatility. The LSTM model further confirms that including sentiment and policy variables can improve the accuracy of volatility prediction. The study also confirms the effectiveness of HAR and LSTM models in predicting educational stock price volatility, as well as the usefulness of sentiment and policy variables in improving prediction accuracy. However, more data sources and external factors should be considered in future research to further improve the reliability and generalizability of the results.

This paper has the following contribution: *(i)* we establish the educational firm's stock volatility index in China; *(ii)* we conduct a dictionary-based sentiment analysis to determine the sentiment index by using the posts in the education area; *(iii)* the article examines both the impacts of educational policy and investor sentiment on education industry's volatility; *(iv)* Consider HAR model and LSTM model to better predict the education firms' price volatility. These findings carry several practical implications, particularly for investors, education-listed companies, and policymakers. Investors could consider various factors including policy changes and company fundamentals for informed decision-making, while education-listed companies could enhance transparency through improved information disclosure and robust risk management systems. Additionally, policymakers can ensure the consistency and stability of education policies and establish effective communication mechanisms to reduce policy uncertainty in the market.

The remainder of the article is organized as follows. Section 2 is the literature review. Section 3 presents the data descriptive. Section 4 shows the methodology design of the HAR model and LSTM model in our article. Section 5 presents the evaluation indicator. Section 6 illustrates the results analysis. Section 7 provides the robustness test results. Finally, we summarize the conclusion in the last Section.

## 2 Literature review

### 2.1 HAR model and LSTM models in predicting stock price volatility

The Heterogeneous AutoRegressive (HAR) model and Long Short-Term Memory (LSTM) model have been widely used to predict stock price volatility. The HAR model has been shown to provide more accurate predictions than traditional models by considering not only the current volatility but also long and short-term dependencies. For instance, Chen et al. [9] compared the HAR model with other models and found that the HAR model is more accurate in predicting volatility. The LSTM model, on the other hand, is a type of artificial neural network that can capture long-term dependencies better than other traditional models. For example, Liu and Lv [10] showed that the LSTM model can produce more accurate predictions than other models.

However, few studies have attempted to combine the HAR and LSTM models in predicting stock price volatility. Li et al. [11] compared the performance of the two models and found that the combined method can improve the accuracy of volatility prediction in the Chinese stock market. Similarly, Zhu et al. [12] found that the combination of HAR and LSTM models can provide better predictions in the Hong Kong stock market. These findings suggest that using both the HAR and LSTM models together can improve the accuracy of stock price volatility predictions. However, the above works do not consider the education firm's stock price prediction and the impact of investor sentiment and policies.

### 2.2 Sentiment index and stock price volatility, especially in education

The sentiment index refers to the measurement of market participants' emotions or feelings regarding the stock market. Numerous studies have shown that the sentiment index has a significant impact on stock price volatility. For example, Baker and Wurgler [13] found that the sentiment index can account for a substantial amount of stock market fluctuations. In the context of education, the sentiment index can play an important role in predicting the volatility of education stocks due to the specific nature of the education industry.

There are currently limited studies that explore the relationship between sentiment index and educational stock market volatility. One study that focused on this relationship is Liu and Su [14], who found that the sentiment index can predict the volatility of Chinese education stocks. Similarly, Liu et al. [15] found that the sentiment index can predict the volatility of the US education stock market. These studies suggest that the sentiment index is a valuable predictor of educational stock market volatility.

### 2.3 The impact of policies on stock price volatility, especially in education

In addition to the sentiment index, policies can also have a significant impact on stock price volatility. This is especially true in the context of the education industry, where government policies can influence the performance of education-related companies. For example, Wang et al. [16] found that policies related to government financial support significantly impact the performance of Chinese education companies.

Few studies have specifically explored the impact of policies on educational stock market volatility [17,18]. However, one study by Jiang and Ye [19] examined the influence of policies on the performance of Chinese education stocks and found that policies related to education reform, research and development, and financial support all have a significant impact on the performance of these companies. This suggests that policies related to education can significantly impact the performance of educational stocks and therefore should be considered when predicting their volatility.

### 2.4 Exploring the research area of stock price volatility prediction, especially in education

The study of stock price volatility prediction is essential for investors and policymakers. However, most of the existing research has focused on the financial sector, with limited studies exploring educational stock market volatility. This knowledge gap highlights the need for further research in this area.

One study that examined educational stock market volatility prediction is Li et al. [20], who used the Wavelet Transform Analysis and GARCH-MIDAS model to predict the volatility of Chinese education stocks. They found that the GARCH-MIDAS model was more accurate than other models. Similarly, Cai and Zhang [21] applied the EGARCH model to predict the volatility of Chinese education stocks and found that the model was an effective predictor. Finally, despite limited research in educational stock price volatility prediction, existing studies suggest that the GARCH-MIDAS and EGARCH models can effectively predict volatility.

In conclusion, the literature suggests that using a combination of HAR and LSTM models can improve the accuracy of predicting educational stock price volatility. Both the sentiment index and policies can significantly impact educational stock price volatility, and further research is needed to explore this area.

## 3 Data description

Nine listed companies are selected from the Shenzhen Stock Exchange and Shanghai Stock Exchange, which are publicly traded on the stock market, and their 5-min stock trading data from January 5, 2015, to October 20, 2022, are collected, including closing prices, trading volumes, and other indicators. The selected companies are Baiyang Investment Group(002696), Shenzhen Kingsun Science(300235), Beijing Kaiwen Education(002695), Suzhou Kingwood(300192), My Gym(002621), Vtron Group(002308), ST(300089), Xin Nanyang(600661), Offcn Education(002607). We chose these samples for the reasons as follows. Firstly, these companies represent a wide range of sub-sectors inside the Chinese education system, including education technology, private education, and vocational training. Because of this diversity, the research is able to get a more complete picture of the drivers and volatility of the sector. Secondly, the companies selected are active participants in the stock markets. The availability of 5-min trading data ensures that the study captures intra-day volatility, which can be influenced by immediate market sentiments and policy announcements [25].

A weighted average method is utilized to construct an educational index volatility for prediction, based on the trading data of these companies. Each trading day includes 48 trading data points, and a total of 819,072 5-min trading data points are collected for the nine companies.

To further determine the daily sentimental index in the educational area, we have collected the interactive posts from East Money Information and Snowball and analyzed their sentiment index using the text analysis framework.

The dataset includes a mix of continuous and categorical variables. Continuous variables include dvol, wvol, mvol, and wsentindex. The related policy variables include COVID-19 and six policy-related variables (Policy 1 through Policy 6), which take on binary values (0 or 1) and reflect the implementation of various policies in response to the COVID-19 pandemic. There are many crucial educational policies announced in recent years, such as Vocational School School-Enterprise Cooperation Promotion Measures, and Double reduction policy, which impact the listing education firms dramatically. The article also shows the details about six different policies in Table 1.

**Table 1:** Notation about important policy.

Variables	Date	Policy	Key point
Policy 1	2018-02-23	Vocational School School-Enterprise Cooperation Promotion Measures	Improve the important role of enterprises in the implementation of vocational education, promote the formation of integration of production and education, and school-enterprise cooperation
Policy 2	2018-08-22	Opinions on Regulating the Development of Off-campus Training Institutions	Strengthen the daily supervision of off-campus training institutions; standardize the order of the off-campus training market; reduce the excessive extracurricular burden of students
Policy 3	2019-02-13	National Vocational Education Reform Implementation Plan	Increase the income of technical and skilled talents; promote the high-quality development of secondary and higher vocational education; Construct national standards for vocational education
Policy 4	2019-07-15	Implementation opinions on standardizing off-campus online training	Strengthen customer information and data security protection; implement the record review system; obtain ICP record qualification; Educational content is healthy
Policy 5	2021-07-24	Double reduction policy	Further reduce the burden of homework and off-campus training for students at the stage of compulsory education; education institutions related to subject training are prohibited from going public for financing.
Policy 6	2022-04-20	China Vocational Education Law	Highlight the role of vocational education; clarify the definition of vocational education; improve the management of vocational education.

And stock market indexes are applied as control variables including, Government bond Yield to Maturity (to make the data more stable, we use its logarithm, ln_bond. The same below.), SSE Composite Index (ln_sindex), CSI 500 INDEX (ln_zindex), S&P 500 (ln_SP500), Dow Jones Industrial Average (ln_DJI), ln_IXIC, VIX CBOE S&P 500 Volatility Index (ln_VIX), and CBOE Gold ETF Volatility Index (ln_GVZ).

Table 2 displays descriptive statistics for a dataset containing 1809 observations of various economic and policy-related variables. The mean values for the variables range from 0.15312 for Policy1 to 12.5739 for mvol, while standard deviations range from 0.0586 for Policy6 to 15.6626 for wsentindex. The minimum and maximum values for each variable are also provided. These statistics can be used to identify any outliers or unusual observations, as well as to compare the distributions of different variables. However, they do not provide any information about the relationships between variables or any causal effect of policy interventions on economic outcomes, which would require additional analyses such as regression models or randomized controlled trials.

**Table 2:** Descriptive statistics of variables.

Variable	Mean	Std	Min	Max
dvol	12.5369	12.7678	0.9659	142.0451
wvol	12.5657	10.7434	1.4970	106.0867
mvol	12.5739	8.9414	2.3747	56.5618
rvd	12.5736	12.9385	0.9659	142.0451
rvw	12.5672	10.7660	1.4970	106.0867
rvm	12.5770	8.9412	2.3747	56.5618
sentindex	−0.6062	0.8474	−3.8067	3.7208
dsentindex	−6.5450	17.2074	−200.7820	141.3540
wsentindex	−6.8297	15.6626	−199.9975	96.3415
msentindex	−6.8529	13.8529	−121.7070	58.4333
lagsentindex	−0.6064	0.8444	−3.8067	3.7208
Covid	0.3571	0.4793	0.0000	1.0000
Policy1	0.5976	0.4905	0.0000	1.0000
Policy2	0.5312	0.4992	0.0000	1.0000
Policy3	0.4721	0.4994	0.0000	1.0000
Policy4	0.4174	0.4933	0.0000	1.0000
Policy5	0.1531	0.3602	0.0000	1.0000
Policy6	0.0586	0.2349	0.0000	1.0000
ln_bond	0.9170	0.2040	0.1137	1.3351
ln_sindex	8.0711	0.1106	7.8097	8.5499
ln_zindex	8.7153	0.1572	8.2987	9.3541
ln_SP500	7.9572	0.2620	7.5280	8.4750
ln_DJI	10.1043	0.2353	9.6593	10.5132
ln_IXIC	8.9732	0.3782	8.3809	9.6839
ln_VIX	2.8483	0.3592	2.2127	4.4151
ln_GVZ	2.7330	0.2597	2.1838	3.8914

**Fig. 1:**
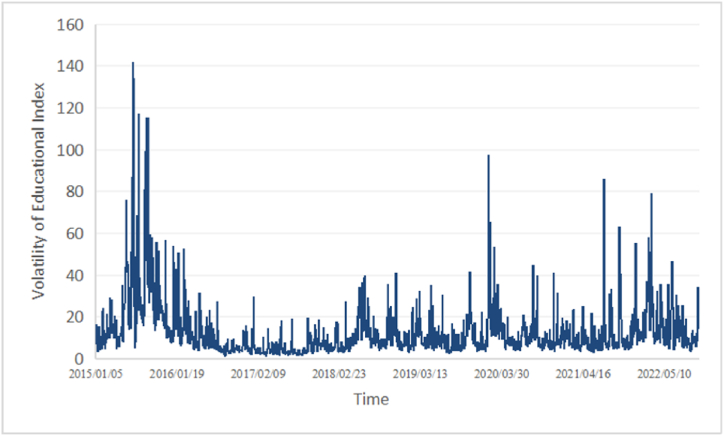
Daily price volatility dynamics of educational index.

**Fig. 2:**
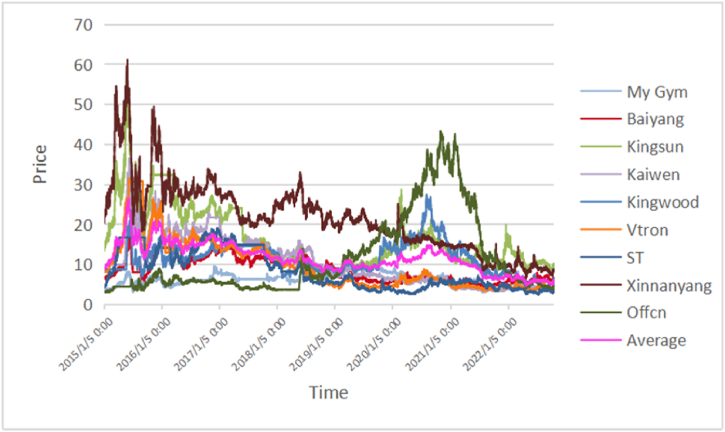
Five minutes high-frequency price dynamics of educational companies.

Fig. 1 shows the average daily price volatility dynamics of chosen educational stocks and Fig. 2 shows the 5-min high-frequency price dynamics of chosen educational companies and their average 5-min high-frequency price.

## 4 Method

We conducted the dictionary-based sentiment analysis to determine the sentiment index by using the posts we collected from East Money Information and Snowball. We have also selected policies that may affect the educational stock market as the policy index. Then we involve both the sentiment index and policy index into two groups of models, HAR models and LSTM models, to identify whether they would affect the volatility. HAR models are applied to determine each variable's significance in affecting the volatility. Four HAR models have been applied in this research. HAR-RV serves as the base model for predicting the realized volatility (RV) of the educational index. It provides a fundamental understanding of the volatility patterns without external modifiers, offering a baseline for comparison [6]. Considering educational indices may be significantly influenced by public sentiment or attitudes toward education, which could arise from media reports, social trends, or academic achievements, we involve the sentiment index and construct the model, HAR-RV-S. It helps to assess how these factors sway volatility beyond standard market variables [22]. Then HAR-RV-P includes the impact of policy changes on volatility. Educational indices can be sensitive to government policies, regulatory changes, or funding adjustments. By modeling this separately, the research can isolate the specific contribution of policy changes to volatility, which is crucial for understanding how policy shifts directly affect the educational sector [23]. By combining both sentiment and policy factors into one model (HAR-RV-SP), we analyze the interactive effects of these variables on volatility. This is particularly relevant in fields like education, where policy decisions often generate significant public reaction, and vice versa, public sentiment can drive policy changes. Understanding how these factors influence each other and the index collectively can provide deeper insights [24]. Each variant of the LSTM model is tailored to incorporate different factors (like Realized Volatility, Sentiment, and Policy), similar to the HAR models but with the added complexity and learning capabilities of neural networks. The result would be more convincing if similar results were obtained in both HAR and LSTM models [25].

### 4.1 Dictionary-based sentiment analysis algorithm for educational companies

The analysis of stock-market-related news, comments on social media, and reports sentiment tendency could be significant for predicting the volatility of stock price because investors' investment can be affected by surrounding news and other investors’ posts. We collected comments related to educational companies, education-related news, and educational policies to construct the sentiment index of educational companies.

#### 4.1.1 Dictionary

Under existing financial dictionaries [26,27], we extract both positive and negative words from the stock market which has been listed in Table 3. These words are used to decide the sentiment of the news, comments, and policies collected. Then the number of positive news, comments, and policies is calculated, so as the negative.

**Table 3:** Emotional seed words in the financial field.

Emotion	Samples								
Positive	扭转	看好	反弹	安稳	爆发	暴涨	飙升	激增	… …
Negative	暗淡	负债	贬值	崩盘	暴跌	欠佳	赤字	惨淡	… …
Examples of words in formal and informal sentiment dictionaries									
In formal sentiment dictionary									
Negative									
风险	亏损	违反	损害	舞弊	严重	约束	手段	坏账	负担
越权	不道德	损毁	异常	谴责	严峻	萎靡	困顿	失利	守旧
不健全	仿造	倒闭	侮辱	压制	冒进	刁难	危害	压迫	低迷
Positive									
平稳	崛起	精神	和谐	突出	合格	力争	透明	成熟	迅速
倾心	保密	清晰	积极性	严正	丰硕	乐观	从优	信誉	充实
不屈	威信	完备	创新	勇气	飙升	富余	干劲	庆祝	强悍
Informal sentiment dictionary									
Negative									
垃圾	下跌	回调	割肉	套牢	风险	减持	抛售	可悲	低迷
向下	跌破	无耻	狗屎	利空	困顿	可笑	跳空	倒霉	赔钱
烂股	小人	绝望	卑鄙	压制	不值	草包	担心	丢脸	烦心
Positive									
涨停	崛起	胜利	献花	发财	暴涨	战斗机	稳赚	过瘾	幸运
黑马	赚翻天	爽歪歪	止跌	恭喜	开心	舒服	漂亮	牛股	完美
赚大	期待	好样	创新	勇气	神奇	明智	成功	飙升	支持

#### 4.1.2 Sentiment index

The dictionary-based sentiment analysis method could be used to decide the sentiment polarity of each comment, news, or policy. But each day's educational sentiment index changes with time so it is essential to calculate each day's sentimental fluctuation to determine the sentiment index. The sentiment index is a way to depict the overall trend of an event and the public mood. Equation (1) defines the sentiment index *sent*_*t*_.

(1) $$sent_{t}=\ln\frac{1+num_{t,pos}}{1+num_{t,neg}}$$

where $num_{t,pos}$ is the total number of positive news, comments and policies on trading day t and $num_{t,neg}$ is the number of negative news, comments and policies on trading day t. Sent_t_ is available to predict and explain the volatility of the sentiment index.

### 4.2 HAR models

To predict the volatility of the educational index, we use the 5-min trading data of listed educational companies to construct the education index. We build a HAR-RV model to predict the volatility of the educational index and further involve the sentimental analysis in the HAR-RV-S model and policy impact in the HAR-RV-P model.

#### 4.2.1 HAR-RV model

Generally, the volatility of the financial market is measured by the realized volatility (RV) proposed by Andersen and Bollerslev [28]. According to Andersen and Bollerslev's calculation method of the realized volatility, we divided a trading day t's trading data i to M sections and used P_t,i_ to denote the ith closing price (i = 1, 2, …, M). Given the logarithmic rate of return for the ith period in trading day t is $r_{t,i}$, $r_{t,i}=100*\left(\ln\;P_{t,i}-\ln\;P_{t,i-1}\right)$, then the RV in trading day *t* could be expressed as Equation (2).

(2) $$RV_{t}=\sum_{i=1}^{M}r_{t,i}^{2}$$

Therefore, the weekly realized return and the monthly realized return could be respectively denoted as Equations (3), (4).

(3) $$RV_{t}^{w}=\frac{RV_{t}^{d}+RV_{t-1}^{d}+RV_{t-2}^{d}+RV_{t-3}^{d}+RV_{t-4}^{d}}{5}$$

(4) $$RV_{t}^{m}=\frac{RV_{t}^{d}+RV_{t-1}^{d}+RV_{t-2}^{d}+\cdots +RV_{t-19}^{d}}{20}$$

According to the “Heterogeneous Market Hypothesis” [29], Corsi [6] proposed that participants in the heterogeneous market may accept different prices, resulting in trading and market fluctuation. He divided the market fluctuation into the short-term, mid-term and long-term. The short-term fluctuation is caused by short-term investors' trading with the reference to daily trading data; the mid-term is due to investors' trading activity with the weekly data; and the long-term fluctuation is based on monthly data. In accordance with the market volatility's long memory feature, Corsi built a volatility prediction model, HAR-RV model, which is defined as Equation (5).

(5) $${\bar{RV}}_{t+H}^{d}=c+\alpha_{1}RV_{t}^{d}+\alpha_{2}RV_{t}^{w}+\alpha_{3}RV_{t}^{m}+\varepsilon_{t+H}$$

where ${\bar{RV}}_{t+H}^{d}=\left(RV_{t+1}^{d}+RV_{t+2}^{d}+\cdots +RV_{t+H}^{d}\right)/H$ (H = 1, 2, …). When H = 1, it represents the next day's RV; when H = 5, it refers to the next week's RV; and when H = 22, it refers to the next month's RV. And $RV_{t}^{d}$, $RV_{t}^{w}$, $RV_{t}^{m}$ denotes period t's daily realized volatility, weekly realized volatility and monthly realized volatility. This model mainly reflects that the volatility of the market is a complex mixture with various combined fluctuation components and it is the outcome of trading activities of short-term, mid-term and long-term investors.

We calculate the weighted average of nine companies’ daily RV, weekly RV and monthly RV and include them in the HAR-RV model to predict the volatility. And other HAR models in this research also apply similar analysis methods.

#### 4.2.2 HAR-RV-S model

Traditional finance supports that the trend of stock depends on economic data and technical indicators. However, some empirical research indicates there is irrational trading in the stock market, especially in China's A-share where private investors dominate the market. Some foreign researchers have proposed relevant theories and conducted in-depth research on the effect of investors' activities and sentiments on the stock market. Affected by investors' “animal spirit” which causes the price to deviate from the fundamentals. It was shown that the sentiment will create noisy trading and over-fluctuation. The question is not whether investors' sentiment will affect the evaluation of stock, but how to measure their sentiment and qualify their influence on the stock.

Some scholars have detected the influence of personal sentiment distribution on the stock market to realize the measurement of sentiment and to highlight the significance of private investors. They used linear regression and SVR to analyze the data collected from Twitter and proved that the prediction of stock volatility could be improved by the sentimental distribution including the stock's future interest and RV. Therefore, it could be concluded the sentiment index would affect the prediction of a stock's volatility.

We calculate the sentiment index of China's educational companies and include it in the HAR model to build the HAR-RV-S model, which is showed in Equation (6).

(6) $${\bar{RV}}_{t+H}^{d}=c+\alpha_{1}RV_{t}^{d}+\alpha_{2}RV_{t}^{w}+\alpha_{3}RV_{t}^{m}+\alpha_{4}sent_{t}+\varepsilon_{t+H}$$

#### 4.2.3 HAR-RV-P model

Policies' release and uncertainty will also influence both microeconomic and real economy. However, most research on the correlation between the stock market's volatility and policies mainly studies the influence of specific policies. There is limited research on the effect of economic policies' uncertainty on the stock market's volatility.

It is of certainty that educational policies will affect the fluctuation of stock prices. For example, when the Double-reduction policy was released, the stock price of all educational companies in China fell dramatically. The uncertainty of such policies has also a great influence on the forecast of stock price volatility. Therefore, the policy factor is also included in the HAR model for prediction by constructing the HAR-RV-P model in Equation (7). The policy factor is indicated as *P*_*i*_.

(7) $${\bar{RV}}_{t+H}^{d}=c+\alpha_{1}RV_{t}^{d}+\alpha_{2}RV_{t}^{w}+\alpha_{3}RV_{t}^{m}+\sum \beta_{i}P_{i}+\varepsilon_{t+H}$$

#### 4.2.4 HAR-RV-SP model

Including both sentiment index and policy factors in the HAR model, we construct the HAR-RV-SP model to forecast the volatility of the education index. The model is expressed as Equation (8).

(8) $${\bar{RV}}_{t+H}^{d}=c+\alpha_{1}RV_{t}^{d}+\alpha_{2}RV_{t}^{w}+\alpha_{3}RV_{t}^{m}+\alpha_{4}sent_{t}+\sum \beta_{i}P_{i}+\varepsilon_{t+H}$$

### 4.3 LSTM model

Long Short-Term Memory (LSTM) is a distinct type of recurrent neural network (RNN) model that was introduced by Hochreiter and Schmidhuber. It is demonstrated to perform better than the random prediction method with a higher accuracy in stock market prediction [30,31], and it could be available to determine factors’ effect on stock market prediction. Lakshminarayanan and McCrae [32] have found that crude oil and gold prices have some impact on stock price prediction. LSTM models incorporated with sentiment data also produce reasonable and good results in stock market prediction [33,34].

**Fig. 3:**
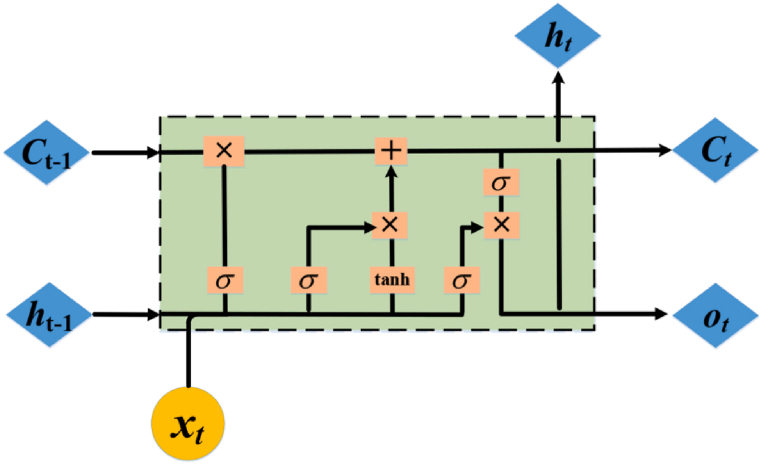
General structure and recurrent module display of LSTM.

With the basic framework of typical RNNs, LSTM employs a different approach to computing hidden states. LSTM was developed to solve the problems of information flooding (short-term memory) and gradient vanishing or exploding during training that plague traditional RNNs. Compared to the architecture of traditional RNN units, LSTM innovatively introduced a control gate structure, enabling the network module to efficiently handle long-term dependencies in time series, and facilitating the efficient transmission of historical temporal signals. Furthermore, unlike traditional deep learning methods, LSTM utilizes historical sequential data to reflect historical information, with output results being determined by both current and historical inputs. Therefore, for time series prediction problems, LSTM has unique advantages in terms of constructing time units. The LSTM neural network structure and its recurrent module are presented in Fig. 3 below.

The ingenuity of LSTM lies in the incorporation of multiple gating mechanisms, with three square-shaped gates–namely, the forget gate, input gate, and output gate–within the recurrent module shown in the figure. These gates selectively control the flow of information by varying the self-recurrent weights, regulating how much information from the previous unit can pass through and which information from the current unit can be transmitted to the next unit. The LSTM recurrent module is characterized by the following calculation formulae. equations (10), (11), (12), (13), (14), (9) demonstrate the input and output of a single time step of the LSTM model. The symbol $x_{t}$ represents the input sequence, while $h_{t-1}$ and $c_{t-1}$ denote the input from the previous LSTM cell, and $o_{t}$ is the output at the current time step. In addition, LSTM generates and utilizes cell states $c_{t}$, $h_{t}$ for the next LSTM cell.

(9) $$\text{Forget}\;\text{gate}:f_{t}=\sigma \left(W_{f}h_{t-1}+U_{f}x_{t}+b_{f}\right)$$

(10) $$\text{Input}\;\text{gate}:i_{t}=\sigma \left(W_{i}h_{t-1}+U_{i}x_{t}+b_{i}\right)$$

(11) $$\text{Output}\;\text{gate}:f_{0}=\sigma \left(W_{0}h_{t-1}+U_{0}x_{t}+b_{0}\right)$$

(12) $$\text{Memory}\;\text{cell}\;\text{candidate}:{\tilde{c}}_{t}=\tanh\left(Wh_{t-1}+Ux_{t}+b\right)$$

(13) $$\text{Memory}\;\text{cell}:c_{t}=f_{t}\circ c_{t-1}+i_{t}\circ {\tilde{c}}_{t}$$

(14) $$\text{Shadow}\;\text{state}:h_{t}=o_{t}\circ \tanh\left(c_{t}\right)$$

Cell output: $y_{t}=h_{t}$, W and U are weight matrices, while residual sum σ is the activation function.

We will compare the prediction results of the LSTM model with those of the HAR model to investigate the influence of different factors on the prediction performance. We have constructed two groups of LSTM models. The first group includes LSTM-RV, LSTM-RV-S, LSTM-RV-P, LSTM-RV-SP and the second group involves stork market indexes (SMI) based on the first group.

#### 4.3.1 LSTM-RV models

The daily volatility, weekly volatility and monthly volatility obtained by HAR-RV model with a lag of one day was taken as input, while the current daily volatility, weekly volatility and monthly volatility were respectively taken as output for prediction, and the LSTM-RV model was constructed.

The LSTM-RV-S model is constructed by including the sentiment index into the LSTM-RV model as additional input variables to separately predict the current day's daily, weekly, and monthly volatility.

The LSTM-RV-P model is constructed by including educational policy factors into the LSTM-RV model as additional input variables to separately predict the current day's daily, weekly, and monthly volatility.

Including both sentiment index and educational policies into the LSTM-RV model, the LSTM-RV-SP is built to forecast the educational index volatility. And results achieved by different models are compared.

#### 4.3.2 LSTM-RV-SMI models

Considering the impacts of stock market indexes like the S&P 500, we involve the stock market indexes into LSTM-RV models respectively and obtain LSTM-RV-SMI models (LSTM-RV-SMI, LSTM-RV-S-SMI, LSTM-RV-P-SMI and LSTM-RV-SP-SMI).

## 5 Evaluation indicator

Both in-sample and out-of-sample predictions are conducted using the above models, and their performance is evaluated by using different indicators. The entire dataset is split into an 90 % in-sample dataset and a 10 % out-of-sample dataset. Commonly, R-squared and adjusted R-squared are used to evaluate regression models. Performance metrics that are used most frequently to evaluate the prediction include Root Mean Square Error (RMSE), Mean Absolute Percentage Error (MAPE), Mean Absolute Error (MAE), Mean Square Error (MSE) and accuracy [35,36]. Patil et al. [37] have use MAE, MAPE and RMSE to compare the performance of graph-based models using deep learning neural networks or machine learning and other traditional and statistical models for stock prediction. Banerjee et al. [38] applied them to evaluate the performance of machine learning and statistic models in price forecasting of vegetables.

In the evaluation of in-sample predictions, R-squared and adjusted R-squared are used to measure the explanatory power of the model. R-squared = SSR/TSS = 1-RSS/TSS, where TSS is the total sum of squares before conducting the regression analysis, RSS is the residual sum of squares, which is the variance that the regression model cannot explain, and SSR is the sum of squares explained by the regression model. It can also be expressed as Equation (15).

(15) $$R^{2}=1-\frac{\sum {\left(RV_{t}-\hat{RV_{t}}\right)}^{2}}{\sum {\left(RV_{t}-\bar{RV_{t}}\right)}^{2}}$$

Adj-R^2^ is expressed as equation (16), where n is the number of observations and p is the input.

(16) $$R_{adj}^{2}=1-\frac{\sum {\left(RV_{t}-\hat{RV_{t}}\right)}^{2}}{\sum {\left(RV_{t}-\bar{RV_{t}}\right)}^{2}}\left(\frac{n-1}{n-p-1}\right)$$

In the field of out-of-sample prediction, the evaluation of the loss function is an important method for assessing the predictive performance of models. A smaller value of the loss function indicates a better predictive performance of the model. Three loss functions have been chosen to measure the performance of our models, including Mean Absolute Error (MAE), Mean Absolute Percentage Error (MAPE), and Root Mean Squared Error (RMSE). Calculating the value of the loss function and comparing the prediction loss of each model can help us evaluate the quality of the predictive models. These three loss functions are defined as Equations (17), (18), (19).

(17) $$MAE=\frac{1}{n}\sum \left|RV_{t}-\hat{RV_{t}}\right|$$

(18) $$MAPE=\frac{1}{n}\sum \frac{\left|RV_{t}-\hat{RV_{t}}\right|}{RV_{t}}$$

(19) $$RMSE=\sqrt{\frac{1}{n}{\sum \left(RV_{t}-\hat{RV_{t}}\right)}^{2}}$$

## 6 Result analysis

We used the Ordinary Least Squares (OLS) regression to estimate the coefficient of HAR models and to figure out whether each independent variable's impact is significant on the educational stock volatility. The sentiment index significantly affects the 1-day and 1-week stock price volatility, while the policy index affects the 1-month stock price volatility significantly. Then we applied LSTM to further test their effects in forecasting the stock price volatility. Results show that the sentiment index and policy index could affect the accuracy of stock price volatility prediction.

### 6.1 OLS regression

In this section, we employ OLS regression to estimate the parameters of four Heterogeneous Autoregressive (HAR) models. By incorporating the sentiment index and policy index into the HAR models, we observe that these two indexes have a notable impact on the volatility. Specifically, we find that the sentiment index has a significant influence on the daily and weekly volatility, while most policy indexes significantly affect the monthly volatility only. Table 4 presents the estimated parameters for the HAR-RV, HAR-RV-S, HAR-RV-P, and HAR-RV-SP models when forecasting volatility of the educational index at three horizons (daily, weekly, and monthly).

**Table 4:** OLS Regression results.

	HAR-RV			HAR-RV-S			HAR-RV-P			HAR-RV-SP		
	**1-day**	**1-week**	**1-month**	**1-day**	**1-week**	**1-month**	**1-day**	**1-week**	**1-month**	**1-day**	**1-week**	**1-month**
dvol	−0.1167∗∗∗	0.0592∗∗∗	0.0033	−0.1311∗∗∗	0.0562∗∗∗	0.0032	−0.1156∗∗∗	0.0601∗∗∗	0.0037	−0.1306∗∗∗	0.0572∗∗∗	0.0036
	(0.0248)	(0.0095)	(0.0024)	(0.0254)	(0.0095)	(0.0024)	(0.0249)	(0.0095)	(0.0024)	(0.0254)	(0.0095)	(0.0024)
wvol	1.1994∗∗∗	0.8241∗∗∗	0.0003	1.1955∗∗∗	0.8381∗∗∗	0.0005	1.2014∗∗∗	0.8261∗∗∗	0.0015	1.1985∗∗∗	0.8395∗∗∗	0.0015
	(0.0395)	(0.0152)	(0.0039)	(0.0395)	(0.0157)	(0.0039)	(0.0396)	(0.0152)	(0.0038)	(0.0396)	(0.0157)	(0.0038)
mvol	−0.0884∗∗	0.1171∗∗∗	0.9869∗∗∗	−0.0842∗∗	0.1137∗∗∗	0.9874∗∗∗	−0.0970∗∗	0.1094∗∗∗	0.9821∗∗∗	−0.0962∗∗	0.1077∗∗∗	0.9824∗∗∗
	(0.0373)	(0.0143)	(0.0037)	(0.0373)	(0.0143)	(0.0037)	(0.0386)	(0.0148)	(0.0037)	(0.0386)	(0.0148)	(0.0038)
dsentindex				−0.0277∗∗∗						−0.0288∗∗∗		
				(0.0105)						(0.0106)		
wsentindex					0.0148∗∗∗						0.0148∗∗∗	
					(0.0045)						(0.0045)	
msentindex						0.0011						0.0005
						(0.0013)						(0.0013)
Covid-19							−0.2245	0.1150	0.4203∗∗∗	0.0103	0.0065	0.4178∗∗∗
							(1.3929)	(0.5332)	(0.1346)	(1.3932)	(0.5328)	(0.1348)
Policy1							0.9401	0.7797∗	0.4308∗∗∗	1.3730	0.5645	0.4245∗∗∗
							(1.0864)	(0.4159)	(0.1049)	(1.0962)	(0.4200)	(0.1063)
Policy2							1.1071	0.9154∗∗	0.2561∗∗	1.0787	0.9331∗∗	0.2564∗∗
							(1.0969)	(0.4199)	(0.1060)	(1.0950)	(0.4188)	(0.1060)
Policy3							−0.1234	−0.3959	−0.3592∗∗∗	−0.1726	−0.3797	−0.3584∗∗∗
							(0.9970)	(0.3816)	(0.0963)	(0.9954)	(0.3806)	(0.0964)
Policy4							−0.0318	0.1681	0.2862∗∗∗	−0.0017	0.1490	0.2857∗∗∗
							(1.0329)	(0.3954)	(0.0998)	(1.0311)	(0.3944)	(0.0998)
Policy5							−1.2426	−0.7869	−0.0628	−0.9678	−0.9310∗	−0.0667
							(1.2507)	(0.4788)	(0.1208)	(1.2527)	(0.4795)	(0.1213)
Policy6							−0.0466	−0.0776	−0.2178∗∗	−0.0633	−0.0752	−0.2183∗∗
							(0.9827)	(0.3762)	(0.0949)	(0.9809)	(0.3751)	(0.0950)
ln_bond	0.5109	−0.4854	−0.4851∗∗∗	0.3674	−0.4280	−0.4803∗∗∗	1.8417	0.5165	−0.1321	1.8924	0.4709	−0.1329
	(1.1484)	(0.4408)	(0.1122)	(1.1478)	(0.4399)	(0.1124)	(1.5521)	(0.5941)	(0.1499)	(1.5495)	(0.5927)	(0.1500)
ln_sindex	−3.5690	−2.0331	1.3368∗∗	−4.4468	−1.4348	1.3868∗∗	−8.7403	−5.2605∗∗	1.1603∗	−9.2703	−4.8364∗	1.1779∗
	(5.5943)	(2.1471)	(0.5468)	(5.5949)	(2.1487)	(0.5499)	(6.6403)	(2.5419)	(0.6414)	(6.6315)	(2.5384)	(0.6433)
ln_zindex	5.1121	4.0860∗∗	0.1890	5.8771	3.6187∗∗	0.1514	10.8372∗	7.9061∗∗∗	0.7704	11.5698∗∗	7.4576∗∗∗	0.7535
	(4.5505)	(1.7465)	(0.4448)	(4.5522)	(1.7473)	(0.4469)	(5.7644)	(2.2066)	(0.5568)	(5.7606)	(2.2049)	(0.5588)
ln_SP500	−5.1876	−7.1887∗∗	−1.4640∗	−6.1749	−6.6523∗	−1.4160	10.6915	3.8174	1.8548	8.9011	4.8283	1.8897
	(9.0465)	(3.4721)	(0.8842)	(9.0392)	(3.4662)	(0.8860)	(14.9229)	(5.7124)	(1.4415)	(14.9113)	(5.7054)	(1.4448)
ln_DJI	8.8447	10.0908∗∗∗	1.9959∗∗	11.0110	8.9900∗∗∗	1.9112∗∗	2.0669	5.5080	0.7986	4.1296	4.4821	0.7647
	(8.7775)	(3.3688)	(0.8579)	(8.8012)	(3.3758)	(0.8635)	(10.1204)	(3.8740)	(0.9776)	(10.1313)	(3.8763)	(0.9820)
ln_IXIC	−1.9942	−1.1988	−0.3433	−2.5190	−0.9645	−0.3295	−9.5130	−6.9326∗∗∗	−2.8350∗∗∗	−10.1519	−6.7010∗∗	−2.8304∗∗∗
	(3.8056)	(1.4606)	(0.3719)	(3.8045)	(1.4582)	(0.3723)	(6.8340)	(2.6160)	(0.6601)	(6.8260)	(2.6099)	(0.6604)
ln_VIX	0.9344	0.1770	0.1279	0.9060	0.1908	0.1293	1.1487	0.2345	0.0626	0.9080	0.3551	0.0664
	(0.8603)	(0.3302)	(0.0841)	(0.8589)	(0.3293)	(0.0841)	(1.0367)	(0.3968)	(0.1001)	(1.0387)	(0.3975)	(0.1007)
ln_GVZ	0.0377	0.1663	−0.2976∗∗	0.0951	0.1405	−0.2994∗∗	−0.0266	0.0510	−0.4689∗∗∗	0.1076	−0.0114	−0.4711∗∗∗
	(1.2171)	(0.4671)	(0.1190)	(1.2153)	(0.4659)	(0.1190)	(1.3948)	(0.5339)	(0.1347)	(1.3932)	(0.5328)	(0.1349)
const	−49.1002	−53.7187∗∗∗	−16.8578∗∗∗	−57.9560	−49.7395∗∗∗	−16.5864∗∗∗	−50.0524	−52.2442∗∗∗	−12.5514∗∗∗	−53.0808	−51.4764∗∗∗	−12.5214∗∗∗
	(38.0230)	(14.5933)	(3.7162)	(38.1075)	(14.6020)	(3.7297)	(41.9759)	(16.0681)	(4.0548)	(41.9169)	(16.0265)	(4.0565)
R-squared	0.7244	0.9414	0.9945	0.7255	0.9417	0.9945	0.7249	0.9418	0.9946	0.7261	0.9421	0.9946
R-squared Adj.	0.7227	0.9410	0.9945	0.7236	0.9413	0.9945	0.7222	0.9412	0.9946	0.7232	0.9415	0.9946

Standard errors in parentheses.∗p < .1, ∗∗p < .05, ∗∗∗p < .01.

In the estimation results of the HAR-RV model, it is observed that the daily, weekly, and monthly volatility are all significantly positive when forecasting 1-week volatility. However, when forecasting 1-day volatility, the daily volatility and monthly volatility are significantly negative, while the weekly volatility is significantly positive. Moreover, for 1-month volatility forecasting, only the monthly volatility is found to be significantly positive. There is not enough significance to forecast 1-month volatility compared with the forecasting of 1-day volatility and 1-week volatility.

The estimation results of HAR-RV-S model show that in 1-day volatility forecasting, the daily volatility and monthly volatility are significantly negative, and the weekly volatility is significantly positive. In 1-week volatility forecasting, all coefficients of daily, weekly, and monthly volatility are significant, and consistent with the result of the HAR-RV model. Next, we examine the coefficients of the sentiment index (sentindex) in the HAR-RV-S model, which is applied using the interaction with senti and volatility to expand the dependence. The result reveals that the sentiment index is significantly negative in 1-day volatility forecasting and significantly positive in 1-week volatility forecasting, but there is not enough significance in 1-month volatility forecasting.

The HAR-RV-P model yields similar estimation results for daily, weekly, and monthly volatility compared to the HAR-RV model. This model also analyzes the impact of COVID-19 and certain policies on volatility. Table 2 shows that most estimation results are significant in 1-month volatility forecasting. The findings reveal that COVID-19 has a significant positive effect on 1-month volatility forecasting. Moreover, the study identifies differences in the coefficients of various policies affecting 1-month forecasting. While some policies have a positive impact, others have a negative effect. For example, Policy 1 - Measures for Promoting the Cooperation between Vocational Schools and Enterprise - has a positive impact as it promotes vocational education. In contrast, the Policy 6 amendment - Vocational Education Law - places more requirements on vocational institutions that could impede their development.

The estimation results of HAR-RV-SP are consistent with those of HAR-RV-S and HAR-RV-P. But both the R-squared value and the Adjusted R-squared value increase. The results present that both the sentiment index and policy index will contribute to the 1-month volatility forecasting.

### 6.2 LSTM forecasting

Through OLS regression, we come to know that both the sentiment index and policy index would affect the volatility. To know whether the prediction accuracy would be improved by considering the sentiment index and policy index, we also perform in-sample and out-of-sample forecasting using the deep learning method, long short-term memory (LSTM). We divide the data into two parts, 90 % as the training data and 10 % as testing data [39]. With data split by 90 % and 10 %, Rana et al. [39] achieve high accuracy in stock price prediction by studying the effects of different activation functions and optimizers on stock price prediction using LSTM networks.

**Fig. 4:**
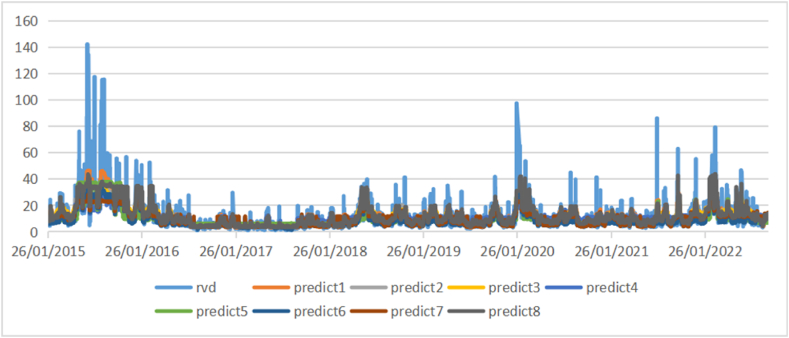
Comparison of actual daily volatility and in-sample predicted daily volatility.

**Fig. 5:**
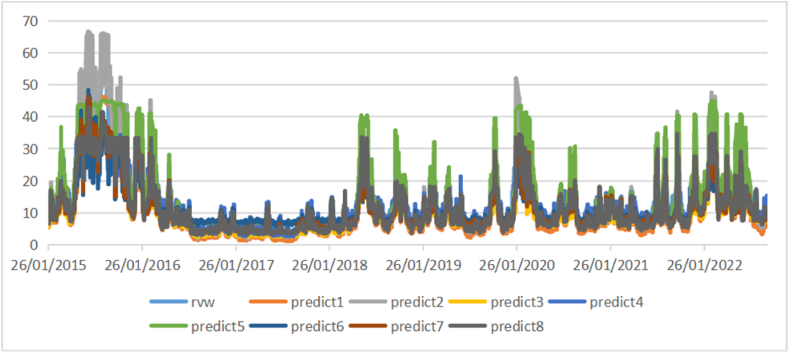
Comparison of actual weekly volatility and in-sample predicted weekly volatility.

**Fig. 6:**
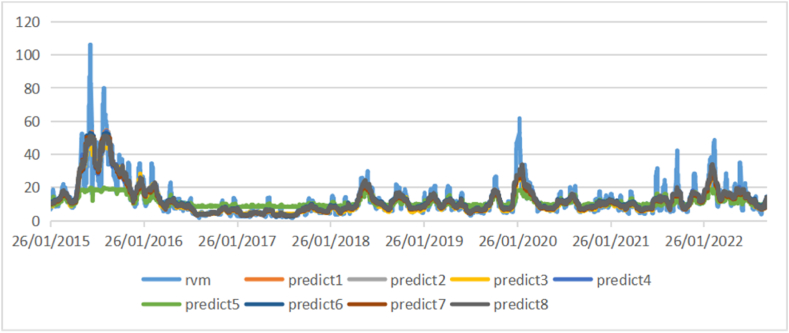
Comparison of actual monthly volatility and in-sample predicted monthly volatility.

Fig. 4, Fig. 5, Fig. 6 show the comparison of the in-sample predicted 1-day, 1-week, and 1-month volatility by eight models as well as the actual volatility. Most predictions have a similar fluctuation trend, although the maximums and minimums are different. Especially in the long-term volatility, the change tendencies of predictions are greatly consistent with that of actual values, with lower values of loss functions.

Table 5, Table 6, Table 7 show the values of loss functions as the out-of-sample prediction evaluation of models. The involvement of a sentiment index or policy index could affect the stock price volatility. For the group of LSTM-RV models, all loss function values decrease for 1-month volatility prediction with sentiment index or policy index being involved. LSTM-RV-P performs the best in the monthly volatility prediction with the smallest loss function values. But for the group of LSTM-RV-SMI models, all loss function values decrease for both 1-day volatility prediction and 1-week volatility prediction. LSTM-RV-P-SMI performs the best in daily volatility prediction and LSTM-RV-SP-SMI performs the best in weekly volatility prediction.

**Table 5:** Prediction evaluation for daily volatility.

Model	R2	ADJ-R2	MAE	MAPE	RMSE
LSTM-RV	0.6002	0.6145	4.2219	0.3159	8.9917
LSTM-RV-S	0.5905	0.5774	4.5718	0.3412	8.1738
LSTM-RV-P	0.5381	0.5381	6.6379	0.4605	11.4683
LSTM-RV-SP	0.5546	0.5449	4.4116	0.3323	8.2146
LSTM-RV-SMI	0.5514	0.5635	5.7381	0.5260	11.1656
LSTM-RV-S-SMI	0.5500	0.5549	5.3465	0.3817	10.3961
LSTM-RV-P-SMI	0.5310	0.5111	4.7015	0.4299	8.8692
LSTM-RV-SP-SMI	0.4872	0.4704	5.5619	0.3918	9.8311

**Table 6:** Prediction evaluation for weekly volatility.

Model	R2	ADJ-R2	MAE	MAPE	RMSE
LSTM-RV	0.8321	0.8220	2.6450	0.1754	4.3936
LSTM-RV-S	0.8568	0.8267	3.0084	0.2307	4.1486
LSTM-RV-P	0.7644	0.7437	2.9721	0.2150	4.6489
LSTM-RV-SP	0.7513	0.7327	3.9748	0.5228	5.4329
LSTM-RV-SMI	0.8156	0.8111	7.3688	0.4260	9.1334
LSTM-RV-S-SMI	0.8338	0.8144	2.9961	0.2359	4.7643
LSTM-RV-P-SMI	0.7352	0.7352	3.9596	0.3153	7.0757
LSTM-RV-SP-SMI	0.7640	0.7640	2.7936	0.2038	5.0432

**Table 7:** Prediction evaluation for monthly volatility.

Model	R2	ADJ-R2	MAE	MAPE	RMSE
LSTM-RV	0.9876	0.9876	1.4061	0.0825	3.1418
LSTM-RV-S	0.9901	0.9901	0.6306	0.0551	0.9952
LSTM-RV-P	0.9912	0.9804	0.5504	0.0464	0.8086
LSTM-RV-SP	0.9804	0.9542	1.0147	0.0756	1.7224
LSTM-RV-SMI	0.9586	0.9228	0.9628	0.0784	1.6631
LSTM-RV-S-SMI	0.9510	0.9510	1.7684	0.1308	2.3380
LSTM-RV-P-SMI	0.9414	0.9144	1.2310	0.0910	1.9655
LSTM-RV-SP-SMI	0.9575	0.9453	1.1080	0.0783	2.1689

**Fig. 7:**
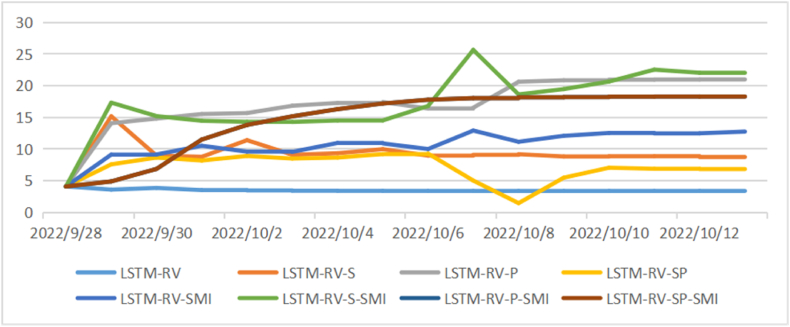
Forecasting for future 15 daily volatility.

**Fig. 8:**
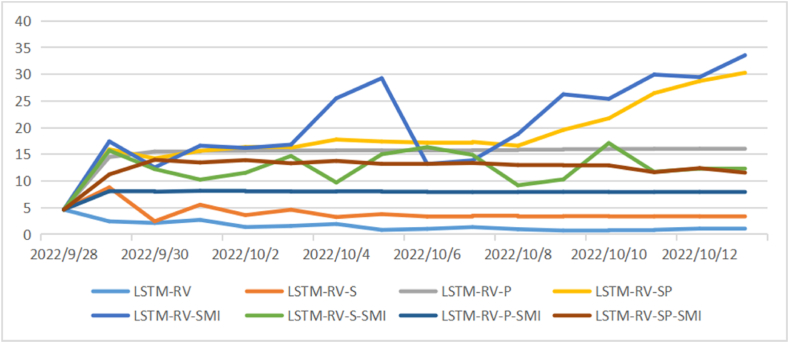
Forecasting for future 15 weekly volatility.

**Fig. 9:**
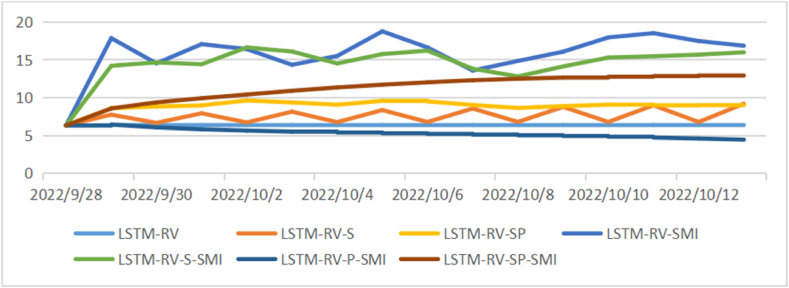
Forecasting for future 15 monthly volatility.

After evaluating the out-of-sample prediction, we apply the LSTM to conduct forecasting for future 15 days’ volatility and make comparisons among those models (see Fig. 7, Fig. 8, Fig. 9). As shown in Fig. 6, the daily volatility is predicted to increase in the next 15 days and the prediction results vary in different models with a range from 3 to 25. The prediction results of weekly volatility in the next 15 days are quite different. Most models have resulting in a range from 0 to 15, while several models get results larger than 30 (see Fig. 7). In comparison, the results in monthly volatility prediction are more stable with a range from 5 to 20 (see Fig. 8).

## 7 Robustness test

To do the robustness check, we conducted regression analysis using different models, rerun the linear regression by cutting the full sample into two parts, and adjusted the LSTM approach.

### 7.1 Regression analysis based on different models

To test each variable's effect on the sock price volatility, we conducted regression analysis by using different models. Those models are OLS, LSDV (least-squares dummy variables), FE (fixed effect), RE (random effect), IVFE (instrumental variable fixed effects) and IVRE (instrumental variable random effects).

As shown in Table 8, Table 9 and Table 10, the sentiment index has a positive significant effect on daily volatility, weekly volatility and monthly volatility. And stock market indexes also have significant impacts on stock price volatility.

**Table 8:** Daily volatility regressions based on different models.

Variable	DOLS	DLSDV	DFE	DRE	DIVFE	DIVRE
dvol	−0.1305∗∗∗	−0.1276∗∗∗	−0.1276∗∗∗	−0.1305∗∗∗	−0.1327∗∗∗	−0.1354∗∗∗
wvol	1.191816∗∗∗	1.1917∗∗∗	1.1917∗∗∗	1.1918∗∗∗	1.1622∗∗∗	1.1629∗∗∗
mvol	−0.0265	−0.0142	−0.0142∗	−0.0265	−0.0131	−0.0248
sentrvm	0.0977	0.1059∗	0.1059∗∗∗	0.0977∗∗∗	0.1005	0.0682
totalvolume	−0.9271∗∗	−1.4877∗∗∗	−1.4877∗∗∗	−0.9271∗∗∗	−1.1032∗∗∗	−0.6576∗∗∗
exchange	−0.5307∗	−0.7891∗∗	−0.7891	−0.5307	−0.3108	−0.0612
bond	−0.5669∗	−0.8557∗∗∗	−0.8557∗∗∗	−0.5669∗	−0.5797∗	−0.3368
sinreturn	−178.7140∗∗∗	−177.0630∗∗∗	−177.0630∗∗∗	−178.7140∗∗∗	−179.3220∗∗∗	−179.6830∗∗∗
zinrturn	28.5416∗∗	29.7545∗∗∗	29.7545∗∗∗	28.5416∗∗∗	9.7443	8.7653
_Icompany_2		−0.0219				
_Icompany_3		−0.1574∗∗∗				
_Icompany_4		0.0979∗				
_Icompany_5		1.6761∗∗∗				
_Icompany_6		−1.5646∗∗∗				
_Icompany_7		−0.4612∗∗∗				
_Icompany_8		0.4365∗∗∗				
_Icompany_9		1.3770∗∗∗				
_cons	19.1683∗∗	30.0475∗∗∗	30.1874∗∗∗	19.1683∗∗∗	20.5056∗∗∗	11.3929∗∗

**Table 9:** Weekly volatility regressions based on different models.

Variable	WOLS	WLSDV	WFE	WRE	WIVFE	WIVRE
dvol	0.0604∗∗∗	0.0616∗∗∗	0.0616∗∗∗	0.0604∗∗∗	0.0467∗∗∗	0.0452∗∗∗
wvol	0.7943∗∗∗	0.7942∗∗∗	0.7942∗∗∗	0.7943∗∗∗	0.7913∗∗∗	0.7920∗∗∗
mvol	0.1658∗∗∗	0.1713∗∗∗	0.1713∗∗∗	0.1658∗∗∗	0.2115∗∗∗	0.2011∗∗∗
sentrvm	0.0397∗∗	0.0431∗∗∗	0.0431∗∗∗	0.0397∗∗∗	0.3311∗∗∗	0.2804∗∗∗
totalvolume	−0.4291∗∗∗	−0.6738∗∗∗	−0.6738∗∗∗	−0.4291∗∗∗	−0.6910∗∗∗	−0.3893∗∗∗
exchange	−0.2168	−0.3266∗	−0.3266∗	−0.2168	−0.5371∗∗	−0.3459
bond	−0.2809∗∗	−0.4072∗∗∗	−0.4072∗∗∗	−0.2809∗∗∗	−0.5507∗∗∗	−0.3832∗∗∗
sinreturn	−21.5701∗∗∗	−20.8226∗∗∗	−20.8226∗∗∗	−21.5701∗∗∗	−22.9219∗∗∗	−22.3608∗∗∗
zinrturn	14.7763∗∗	15.3158∗∗∗	15.3158∗∗∗	14.7763∗∗∗	14.3767∗∗∗	13.8700∗∗∗
_Icompany_2		−0.0498∗∗∗				
_Icompany_3		−0.0876∗∗∗				
_Icompany_4		−0.0284				
_Icompany_5		0.6931∗∗∗				
_Icompany_6		−0.7243∗∗∗				
_Icompany_7		−0.2569∗∗∗				
_Icompany_8		0.1147∗∗				
_Icompany_9		0.5601∗∗∗				
_cons	8.6216∗∗∗	13.3898∗∗∗	13.4086∗∗∗	8.6216∗∗∗	15.6695∗∗∗	9.3142∗∗∗

**Table 10:** Monthly volatility regressions based on different models.

Variable	MOLS	MLSDV	MFE	MRE	MIVFE	MIVRE
dvol	−0.0003	−0.0001	−0.0001	−0.0003	0.0001	−0.0001
wvol	0.0100∗∗	0.0100∗∗	0.0100∗∗∗	0.0100∗∗∗	0.0083∗∗∗	0.0082∗∗∗
mvol	0.9841∗∗∗	0.9845∗∗∗	0.9845∗∗∗	0.9841∗∗∗	0.9881∗∗∗	0.9870∗∗∗
sentrvm	0.0049∗∗	0.0055∗∗∗	0.0055∗∗∗	0.0049∗∗	0.0281∗	0.0220
totalvolume	−0.0587∗∗	−0.0885∗∗∗	−0.0885∗∗∗	−0.0587∗∗∗	−0.0897∗∗∗	−0.0515∗∗∗
exchange	−0.1972∗	−0.2159 ∗∗	−0.2159∗∗∗	−0.1972∗∗∗	−0.1639∗∗	−0.1366∗
bond	−0.0820	−0.0968∗	−0.0968∗∗∗	−0.0820∗∗∗	−0.1061∗∗∗	−0.0863∗∗
sinreturn	−0.2474	−0.1689	−0.1689	−0.2474	−0.8865	−0.8780
zinrturn	5.9852∗∗∗	6.0406∗∗∗	6.0406∗∗∗	5.9852∗∗∗	6.6485∗∗∗	6.5794∗∗∗
_Icompany_2		0.0166∗∗∗				
_Icompany_3		0.0589∗∗∗				
_Icompany_4		0.0293∗∗∗				
_Icompany_5		0.1219∗∗∗				
_Icompany_6		−0.0647∗∗∗				
_Icompany_7		0.0169∗∗				
_Icompany_8		0.0750∗∗∗				
_Icompany_9		0.0855∗∗∗				
_cons	2.4997∗∗	3.0839∗∗∗	3.1204∗∗∗	2.4997∗∗∗	2.8506∗∗∗	2.0275∗∗∗

### 7.2 Robustness test for in-sample regression

Then we rerun the OLS regression to verify the reliability of the forecasting results across different samples. Considering previous studies [[40], [41], [42]], we divided the 1809 samples into two subsets, namely sub-sample 1 (comprising of samples 1 to 904) and sub-sample 2 (comprising of samples 905 to 1809). This would help us understand the robustness of results over time. We conducted an in-sample regression analysis on both sub-sample 1 and sub-sample 2, and the corresponding results are presented in Table 11 and Table 12, respectively. The parameter estimates obtained from both sub-samples are in line with those reported in Table 3, indicating that the forecasting results can be trusted across different samples.

**Table 11:** In-sample regression results on sub-sample 1.

	HAR-RV			HAR-RV-S			HAR-RV-P			HAR-RV-SP		
	1-day	1-week	1-month	1-day	1-week	1-month	1-day	1-week	1-month	1-day	1-week	1-month
dvol	−0.1224∗∗∗	0.0643∗∗∗	0.0040	−0.1602∗∗∗	0.0611∗∗∗	0.0037	−0.1220∗∗∗	0.0647∗∗∗	0.0041	−0.1617∗∗∗	0.0618∗∗∗	0.0039
	(0.0353)	(0.0133)	(0.0034)	(0.0362)	(0.0133)	(0.0034)	(0.0353)	(0.0133)	(0.0033)	(0.0362)	(0.0133)	(0.0033)
wvol	1.1819∗∗∗	0.8231∗∗∗	−0.0020	1.1663∗∗∗	0.8434∗∗∗	−0.0014	1.1874∗∗∗	0.8279∗∗∗	−0.0005	1.1733∗∗∗	0.8453∗∗∗	−0.0002
	(0.0553)	(0.0209)	(0.0053)	(0.0550)	(0.0221)	(0.0053)	(0.0555)	(0.0209)	(0.0053)	(0.0551)	(0.0220)	(0.0053)
mvol	−0.0465	0.1261∗∗∗	0.9878∗∗∗	−0.0363	0.1215∗∗∗	0.9891∗∗∗	−0.0533	0.1200∗∗∗	0.9861∗∗∗	−0.0450	0.1167∗∗∗	0.9870∗∗∗
	(0.0543)	(0.0206)	(0.0052)	(0.0539)	(0.0205)	(0.0053)	(0.0546)	(0.0206)	(0.0052)	(0.0541)	(0.0205)	(0.0053)
dsentindex				−0.0558∗∗∗						−0.0587∗∗∗		
				(0.0138)						(0.0139)		
wsentindex					0.0163∗∗∗						0.0143∗∗	
					(0.0058)						(0.0059)	
msentindex						0.0021						0.0013
						(0.0016)						(0.0016)
Covid-19							−0.0000	−0.0000∗∗∗	−0.0000∗∗∗	−0.0000	0.0000∗∗∗	−0.0000∗∗∗
							(0.0000)	(0.0000)	(0.0000)	(0.0000)	(0.0000)	(0.0000)
Policy1							1.2115	1.1125∗∗	0.3581∗∗∗	1.9604	0.9272∗	0.3424∗∗
							(1.4358)	(0.5407)	(0.1360)	(1.4334)	(0.5446)	(0.1374)
Policy2							1.6579	1.4144∗∗	0.3472∗∗	1.8887	1.3513∗∗	0.3395∗∗
							(1.5191)	(0.5721)	(0.1439)	(1.5059)	(0.5711)	(0.1442)
Policy3							−0.0000	−0.0000∗∗∗	−0.0000∗∗∗	0.0000	−0.0000∗∗	0.0000∗∗∗
							(0.0000)	(0.0000)	(0.0000)	(0.0000)	(0.0000)	(0.0000)
Policy4							−0.0000	−0.0000∗∗	−0.0000∗∗∗	0.0000	−0.0000∗∗∗	0.0000∗∗∗
							(0.0000)	(0.0000)	(0.0000)	(0.0000)	(0.0000)	(0.0000)
Policy5							0.0000	0.0000	0.0000	0.0000	0.0000	0.0000
							(0.0000)	(0.0000)	(0.0000)	(0.0000)	(0.0000)	(0.0000)
Policy6							0.0000	0.0000∗∗∗	0.0000∗∗∗	−0.0000	0.0000∗∗∗	−0.0000∗∗∗
							(0.0000)	(0.0000)	(0.0000)	(0.0000)	(0.0000)	(0.0000)
ln_bond	2.2268	0.6595	−0.6180∗∗	1.5041	0.8126	−0.5981∗∗	4.4405	2.6083∗∗	−0.0746	4.4316	2.5520∗∗	−0.0803
	(2.5464)	(0.9635)	(0.2427)	(2.5310)	(0.9614)	(0.2430)	(3.0180)	(1.1366)	(0.2859)	(2.9898)	(1.1337)	(0.2860)
ln_sindex	−12.1004	−7.5955∗∗	1.1624	−10.9154	−7.7225∗∗	1.1672	−17.4381∗	−12.2445∗∗∗	−0.0815	−17.6347∗	−11.9848∗∗∗	−0.0416
	(8.8199)	(3.3371)	(0.8405)	(8.7497)	(3.3247)	(0.8402)	(9.6716)	(3.6423)	(0.9162)	(9.5813)	(3.6338)	(0.9177)
ln_zindex	11.6404	8.8222∗∗∗	0.6007	11.1508	8.8468∗∗∗	0.5858	17.5689∗∗	13.9885∗∗∗	1.9860∗∗	18.6776∗∗	13.5929∗∗∗	1.9353∗∗
	(7.4123)	(2.8045)	(0.7064)	(7.3502)	(2.7939)	(0.7062)	(8.6173)	(3.2453)	(0.8163)	(8.5408)	(3.2404)	(0.8189)
ln_SP500	21.2324	31.8512	9.3418∗	8.2450	36.1609∗	9.9630∗∗	18.8568	29.8151	8.8318∗	4.7855	33.7066∗	9.2292∗
	(52.6997)	(19.9395)	(5.0222)	(52.3497)	(19.9235)	(5.0423)	(52.7431)	(19.8631)	(4.9964)	(52.3560)	(19.8725)	(5.0213)
ln_DJI	0.6784	−5.9560	−2.5568	3.6911	−6.9676	−2.7190	3.5481	−3.3030	−1.6847	8.6264	−4.6620	−1.8248
	(28.6841)	(10.8529)	(2.7336)	(28.4497)	(10.8178)	(2.7352)	(28.9283)	(10.8944)	(2.7404)	(28.6830)	(10.8786)	(2.7464)
ln_IXIC	−13.6414	−14.5407∗	−3.4578∗	−4.8629	−17.3112∗∗	−3.8472∗∗	−17.4701	−18.1453∗∗	−4.7073∗∗	−11.2514	−19.8356∗∗	−4.8876∗∗
	(19.7241)	(7.4629)	(1.8797)	(19.6763)	(7.5003)	(1.9021)	(20.6989)	(7.7953)	(1.9608)	(20.5581)	(7.8046)	(1.9738)
ln_VIX	1.9610	0.9117	0.4067∗∗	1.5820	1.0210	0.4222∗∗	1.3913	0.3956	0.2476	0.6915	0.5664	0.2637
	(1.8088)	(0.6844)	(0.1724)	(1.7958)	(0.6829)	(0.1727)	(1.8930)	(0.7129)	(0.1793)	(1.8826)	(0.7144)	(0.1805)
ln_GVZ	−0.6384	−0.3023	−0.1054	0.6034	−0.6498	−0.1585	−0.5719	−0.2591	−0.1093	0.6455	−0.5430	−0.1408
	(2.9294)	(1.1084)	(0.2792)	(2.9206)	(1.1111)	(0.2820)	(2.9502)	(1.1111)	(0.2795)	(2.9368)	(1.1141)	(0.2822)
const	−63.0410	−80.0074∗∗∗	−31.7414∗∗∗	−75.0785	−78.1067∗∗∗	−31.4044∗∗∗	−49.9053	−67.9698∗∗∗	−27.8905∗∗∗	−54.6324	−68.2673∗∗∗	−27.8490∗∗∗
	(63.2315)	(23.9244)	(6.0259)	(62.7640)	(23.8432)	(6.0289)	(65.0232)	(24.4878)	(6.1597)	(64.4251)	(24.4205)	(6.1611)
R-squared	0.7618	0.9538	0.9961	0.7661	0.9542	0.9961	0.7623	0.9543	0.9962	0.7670	0.9546	0.9962
R-squared Adj.	0.7588	0.9532	0.9961	0.7629	0.9536	0.9961	0.7588	0.9537	0.9961	0.7633	0.9539	0.9961
No. observations	904	904	904	904	904	904	904	904	904	904	904	904
Standard errors in parentheses.∗p < .1, ∗∗p < .05, ∗∗∗p < .01

**Table 12:** In-sample regression on sub-sample 2.

	HAR-RV			HAR-RV-S			HAR-RV-P			HAR-RV-SP		
	1-day	1-week	1-month	1-day	1-week	1-month	1-day	1-week	1-month	1-day	1-week	1-month
dvol	−0.1084∗∗∗	0.0531∗∗∗	0.0024	−0.0938∗∗∗	0.0497∗∗∗	0.0026	−0.1053∗∗∗	0.0547∗∗∗	0.0028	−0.0908∗∗	0.0513∗∗∗	0.0031
	(0.0352)	(0.0137)	(0.0035)	(0.0355)	(0.0138)	(0.0036)	(0.0353)	(0.0137)	(0.0035)	(0.0356)	(0.0138)	(0.0035)
wvol	1.2313∗∗∗	0.8256∗∗∗	0.0031	1.2258∗∗∗	0.8334∗∗∗	0.0031	1.2359∗∗∗	0.8281∗∗∗	0.0046	1.2302∗∗∗	0.8359∗∗∗	0.0046
	(0.0573)	(0.0224)	(0.0058)	(0.0572)	(0.0226)	(0.0058)	(0.0575)	(0.0224)	(0.0056)	(0.0573)	(0.0226)	(0.0056)
mvol	−0.1479∗∗	0.1299∗∗∗	0.9890∗∗∗	−0.1519∗∗	0.1283∗∗∗	0.9885∗∗∗	−0.1706∗∗	0.1148∗∗∗	0.9726∗∗∗	−0.1754∗∗	0.1125∗∗∗	0.9721∗∗∗
	(0.0636)	(0.0248)	(0.0064)	(0.0634)	(0.0248)	(0.0064)	(0.0699)	(0.0272)	(0.0068)	(0.0697)	(0.0272)	(0.0069)
dsentindex				0.0497∗∗						0.0499∗∗		
				(0.0194)						(0.0195)		
wsentindex					0.0195∗∗						0.0196∗∗	
					(0.0085)						(0.0085)	
msentindex						−0.0017						−0.0021
						(0.0023)						(0.0023)
Covid-19							0.6678	0.4878	0.9326∗∗∗	0.6230	0.4967	0.9303∗∗∗
							(1.7047)	(0.6640)	(0.1667)	(1.6994)	(0.6624)	(0.1668)
Policy1							−9.6876	−11.7874	1.7261	−14.4115	−13.1930	1.9432
							(30.6964)	(11.9564)	(3.0026)	(30.6560)	(11.9431)	(3.0128)
Policy2							−9.6876	−11.7874	1.7261	−14.4115	−13.1930	1.9432
							(30.6964)	(11.9564)	(3.0026)	(30.6560)	(11.9431)	(3.0128)
Policy3							−1.6160	−0.8751∗	−0.2302∗	−1.4542	−0.8170	−0.2357∗
							(1.3402)	(0.5220)	(0.1311)	(1.3376)	(0.5214)	(0.1313)
Policy4							0.2577	0.3775	0.3655∗∗∗	0.0868	0.3087	0.3718∗∗∗
							(0.9828)	(0.3828)	(0.0961)	(0.9820)	(0.3831)	(0.0964)
Policy5							−1.1728	−0.4156	0.3235∗∗	−1.2615	−0.4592	0.3257∗∗
							(1.4341)	(0.5586)	(0.1403)	(1.4300)	(0.5576)	(0.1403)
Policy6							−0.7344	−0.4970	−0.3849∗∗∗	−0.8092	−0.5337	−0.3790∗∗∗
							(1.0079)	(0.3926)	(0.0986)	(1.0051)	(0.3919)	(0.0988)
ln_bond	−0.0045	−0.5800	−0.2542	0.2828	−0.4484	−0.2662	−0.0877	−0.5756	0.0984	0.1823	−0.4386	0.0835
	(1.6963)	(0.6620)	(0.1707)	(1.6948)	(0.6628)	(0.1716)	(1.8992)	(0.7398)	(0.1858)	(1.8962)	(0.7404)	(0.1865)
ln_sindex	12.8373	8.7560∗	1.5962	12.7573	8.8244∗	1.5821	18.7740	13.0320∗∗	6.0576∗∗∗	17.9205	12.8666∗∗	6.0496∗∗∗
	(11.5693)	(4.5147)	(1.1645)	(11.5334)	(4.5039)	(1.1650)	(14.1899)	(5.5270)	(1.3880)	(14.1495)	(5.5142)	(1.3882)
ln_zindex	−0.6215	−1.9064	−0.6825	−1.5377	−2.3513	−0.6510	−1.2655	−2.9339	−3.2982∗∗∗	−1.9194	−3.3136	−3.2574∗∗∗
	(8.4948)	(3.3149)	(0.8551)	(8.4760)	(3.3126)	(0.8565)	(9.9132)	(3.8612)	(0.9697)	(9.8855)	(3.8554)	(0.9709)
ln_SP500	9.3929	0.4579	−0.1567	3.9650	−1.4151	0.0021	40.0049	15.2665	3.4161	35.7128	14.1734	3.5404
	(18.3343)	(7.1546)	(1.8455)	(18.3995)	(7.1834)	(1.8595)	(27.3689)	(10.6604)	(2.6772)	(27.3348)	(10.6452)	(2.6811)
ln_DJI	−4.4885	1.7198	−0.2205	−0.1062	3.1929	−0.3576	−23.1446	−7.3292	−2.4343	−19.0692	−6.1764	−2.5771
	(19.9978)	(7.8038)	(2.0129)	(20.0088)	(7.8111)	(2.0227)	(23.3947)	(9.1124)	(2.2884)	(23.3758)	(9.1041)	(2.2942)
ln_IXIC	−8.3218	−3.0856	0.2536	−6.0546	−2.2375	0.1928	−21.4118∗∗	−9.9686∗∗	−2.8714∗∗∗	−19.4419∗	−9.3958∗∗	−2.9162∗∗∗
	(5.5872)	(2.1803)	(0.5624)	(5.6396)	(2.2059)	(0.5690)	(10.0403)	(3.9108)	(0.9821)	(10.0385)	(3.9092)	(0.9835)
ln_VIX	0.6154	0.4276	0.1147	0.9526	0.5658	0.1023	0.4246	0.3946	0.0449	0.8153	0.5482	0.0277
	(1.2854)	(0.5016)	(0.1294)	(1.2882)	(0.5040)	(0.1306)	(1.4817)	(0.5771)	(0.1449)	(1.4849)	(0.5796)	(0.1462)
ln_GVZ	−0.0491	−0.1785	−0.4316∗∗∗	0.0240	−0.1634	−0.4341∗∗∗	−0.2257	−0.4815	−0.8513∗∗∗	−0.0715	−0.4400	−0.8567∗∗∗
	(1.3896)	(0.5423)	(0.1399)	(1.3856)	(0.5410)	(0.1400)	(1.6421)	(0.6396)	(0.1606)	(1.6380)	(0.6383)	(0.1608)
const	−52.8619	−47.2912	−4.6141	−67.3315	−52.2077	−4.0488	−9.6876	−11.7874	1.7261	−14.4115	−13.1930	1.9432
	(87.1462)	(34.0071)	(8.7718)	(87.0585)	(33.9920)	(8.8103)	(30.6964)	(11.9564)	(3.0026)	(30.6560)	(11.9431)	(3.0128)
R-squared	0.6291	0.9014	0.9861	0.6318	0.9020	0.9861	0.6304	0.9021	0.9869	0.6331	0.9027	0.9870
R-squared Adj.	0.6245	0.9002	0.9860	0.6268	0.9006	0.9860	0.6237	0.9003	0.9867	0.6261	0.9008	0.9867
No. observations	905	905	905	905	905	905	905	905	905	905	905	905

Standard errors in parentheses.∗p < .1, ∗∗p < .05, ∗∗∗p < .01.

Table 13 shows the adjusted squares of OLS regression in both sub-sample 1 and sub-sample 2. In the 1-day volatility estimation, the adjusted squares of sub-sample 1 and sub-sample 2 are quite different and lack robustness. But in 1-week and 1-month volatility estimation, the adjusted R-squares are almost the same between the two sub-samples, indicating that all models are stable in mid-term and long-term volatility forecasting.

**Table 13:** Adjusted squares of sub-sample 1 and sub-sample 2.

	1-day		1-week			1-month
	sub-sample1	sub-sample2	sub-sample1	sub-sample2	sub-sample1	sub-sample2
HAR-RV	0.7588	0.6245	0.9532	0.9002	0.9961	0.9860
HAR-RV-S	0.7629	0.6268	0.9536	0.9006	0.9961	0.9860
HAR-RV-P	0.7588	0.6237	0.9537	0.9003	0.9961	0.9867
HAR-RV-SP	0.7633	0.6261	0.9539	0.9008	0.9961	0.9867

### 7.3 Robustness test for LSTM forecasting

To examine the robustness of LSTM forecasting, we adjust the timesteps value to conduct a new round of forecasting. Table 14 shows that the involvement of the sentiment index or policy index could improve the accuracy of 1-day volatility forecasting in the group of LSTM-RV-SMI models. The effect is more obvious in 1-month volatility forecasting in both LSTM-RV models and LSTM-RV-SMI models. The LSTM-RV-P model performs best in 1-month volatility forecasting with the smallest loss function values which is consistent with section 6.2. LSTM-RV-SP-SMI is also relatively the best one for 1-week volatility prediction.

**Table 14:** LSTM forecasting (1447).

	Model	MAE	MAPE	RMSE
1-day	LSTM-RV	4.5702	0.4309	9.4301
	LSTM-RV-S	7.3011	0.4254	10.3735
	LSTM-RV-P	5.3097	0.4118	10.2217
	LSTM-RV-SP	7.1532	0.5096	10.6521
	LSTM-RV-SMI	5.1566	0.3914	9.5410
	LSTM-RV-S-SMI	5.1983	0.4707	10.0219
	LSTM-RV-P-SMI	5.1528	0.4268	10.4805
	LSTM-RV-SP-SMI	5.2778	0.4060	8.8251
1-week	LSTM-RV	2.5107	0.2005	3.4527
	LSTM-RV-S	3.0913	0.2376	4.2900
	LSTM-RV-P	2.8441	0.2176	4.9277
	LSTM-RV-SP	2.3735	0.2015	3.9610
	LSTM-RV-SMI	4.7169	0.4189	8.1858
	LSTM-RV-S-SMI	5.8010	0.3765	6.9888
	LSTM-RV-P-SMI	7.7122	0.4853	9.6044
	LSTM-RV-SP-SMI	4.1849	0.2800	6.0068
1-month	LSTM-RV	0.8500	0.0725	1.2535
	LSTM-RV-S	0.9033	0.0737	1.4431
	LSTM-RV-P	0.5143	0.0404	0.8609
	LSTM-RV-SP	1.1908	0.0870	2.0427
	LSTM-RV-SMI	5.7752	0.3896	7.6972
	LSTM-RV-S-SMI	1.2740	0.1028	2.0459
	LSTM-RV-P-SMI	1.3700	0.1041	2.0918
	LSTM-RV-SP-SMI	1.5264	0.1237	2.2708

The robustness tests conducted through multiple regression analyses and adjustments to the LSTM model affirm the stability and reliability of the results regarding stock price volatility. Both sentiment and stock market indices significantly affect stock price volatility. Adjustments in the LSTM model's timesteps further confirmed the accuracy of the forecasts, particularly showing that including sentiment or policy indices improves the forecasting of short-term and especially long-term volatility, with the LSTM models tailored to specific volatility durations performing optimally in their respective forecasts.

## 8 Discussion

### 8.1 Theoretical implications

This study mainly makes three theoretical contributions to the existing literature. First, this paper adds to the literature on forecasting financial time series. Our findings highlight that the sentiment index has a significant impact on the daily and weekly volatility of education stock prices, while the policy index has a significant impact on the monthly volatility. In line with prior studies, our findings indicate that sentiment variables have only short-term effects on volatility [43]. Second, this study employs the LSTM model, which is capable of capturing long-term dependencies more effectively. This capability expands upon previous studies by employing the LSTM model for forecasting volatility. Consistent with previous research [44]¸ [45], our study found that the LSTM model demonstrates a significant performance improvement when compared with the HAR model. Thirdly, our study has optimized the model's forecasting performance by incorporating the sentiment index and policy index into the model. This enhancement not only improves the accuracy of volatility predictions but also underscores the importance of investor sentiment and policy uncertainty as factors affecting the volatility of education stock prices.

### 8.2 Practical implications

This study has found that both investor sentiment and policy changes significantly impact the volatility of education stock prices. These findings carry several practical implications, particularly for investors, education-listed companies, and policymakers. First, investors should consider policy changes, investor sentiment, company fundamentals, risk management and other factors and make rational investments. Secondly, publicly listed education companies should enhance their information disclosure practices by promptly and accurately informing investors about the company's operational status and the impact of relevant policies. This will improve the transparency of information. Additionally, these companies should strengthen their risk management practices by establishing a sound risk management system. This will improve their ability to respond to policy changes and market fluctuations. Thirdly, when formulating policies in the education sector, the government should fully consider the consistency and stability of these policies to avoid frequent policy changes that could impact the education stock market. Additionally, a policy communication mechanism can be established to promptly convey policy information to the market, thereby reducing policy uncertainty.

## 9 Conclusion

As an important industry in my country, education enterprises have a lot of room for development, and a large number of investors in the capital market are also eager to conduct investment, which has also attracted many people's attention to the stock price and volatility of education listing enterprises. The impact of sentiment indexes and policy is investigated to explore their effects on educational stock price volatility by HAR model and LSTM prediction in this article.

We establish China's education listing firm's stock volatility index and educational investor sentiment index based on text analysis. Then, the HAR model is conducted to examine the impact of different investors' trading behaviors on education listing enterprises. Based on the stock trading data from January 5, 2015, to October 20, 2022, the findings of this research indicate that the sentiment index significantly influences the daily and weekly fluctuations in educational stock prices, whereas the policy index markedly affects monthly volatility. Additionally, the LSTM model substantiates that integrating sentiment and policy variables enhances the precision of volatility forecasts. This study also verifies the efficacy of HAR and LSTM models in predicting the volatility of educational stock prices and underscores the value of sentiment and policy variables in boosting prediction accuracy. Our findings offers practical implications for investors, education-listed companies, and policymakers, suggesting the adoption of enhanced information disclosure and robust risk management practices to stabilize the education sector's stock market dynamics.

This study has several limitations. Firstly, this study only used data from nine listed education companies. Future research can expand the data sources, include more listed education companies and market data, and improve the representativeness and reliability of the research results. Secondly, this study did not consider the impact of external factors such as the macroeconomic environment and industry development trends. Future research can consider the impact of more external factors and build a more comprehensive education stock price fluctuation prediction model. Thirdly, this study did not analyze how investors with different risk preferences affect education stock price fluctuations. Future research can further analyze investor behavior and provide investors with more accurate investment advice.

## Data availability statement

Data will be made available on request.

## CRediT authorship contribution statement

**Xuefan Li:** Writing – original draft, Visualization, Supervision, Software, Methodology, Investigation, Formal analysis, Conceptualization. **Donghua Li:** Writing – review & editing, Methodology, Investigation, Formal analysis. **Yuxiang Cheng:** Writing – original draft, Supervision, Project administration, Methodology, Data curation. **Wen Li:** Writing – review & editing, Visualization, Investigation.

## Declaration of competing interest

The authors declare that they have no known competing financial interests or personal relationships that could have appeared to influence the work reported in this paper.
